# Inhibition of A549 Lung Cancer Cell Migration and Invasion by *Ent*-Caprolactin C via the Suppression of Transforming Growth Factor-β-Induced Epithelial—Mesenchymal Transition

**DOI:** 10.3390/md19080465

**Published:** 2021-08-19

**Authors:** So Young Kim, Myoung-Sook Shin, Geum Jin Kim, Hyukbean Kwon, Myong Jin Lee, Ah-Reum Han, Joo-Won Nam, Chan-Hun Jung, Ki Sung Kang, Hyukjae Choi

**Affiliations:** 1 College of Pharmacy, Yeungnam University, Gyeongsan-si 38541, Gyeongsangbuk-do, Korea; sososo0305@hanmail.net (S.Y.K.); canta87@ynu.ac.kr (G.J.K.); zero9602@gmail.com (H.K.); jwnam@yu.ac.kr (J.-W.N.); 2 College of Korean Medicine, Gachon University, Seongnam 13120, Gyeonggi-do, Korea; ms.shin@gachon.ac.kr (M.-S.S.); myongene@naver.com (M.J.L.); 3 Research Institute of Cell Culture, Yeungnam University, Gyeongsan-si 38541, Gyeongsangbuk-do, Korea; 4 Advanced Radiation Technology Institute, Korea Atomic Energy Research Institute, Jeongeup-si 56212, Jeollabuk-do, Korea; arhan@kaeri.re.kr; 5 Jeonju AgroBio-Materials Institute, Jeonju-si 54810, Jeollabuk-do, Korea; biohun@gmail.com

**Keywords:** caprolactin C, *ent*-caprolactin C, *Aquimarina* sp., epithelial–mesenchymal transition, A549 human lung cancer cell

## Abstract

The epithelial–mesenchymal transition (EMT) of cancer cells is a crucial process in cancer cell metastasis. An *Aquimarina* sp. MC085 extract was found to inhibit A549 human lung cancer cell invasion, and caprolactin C (**1**), a new natural product, α-amino-ε-caprolactam linked to 3-methyl butanoic acid, was purified through bioactivity-guided isolation of the extract. Furthermore, its enantiomeric compound, *ent*-caprolactin C (**2**), was synthesized. Both **1** and **2** inhibited the invasion and γ-irradiation-induced migration of A549 cells. In transforming growth factor-β (TGF-β)-treated A549 cells, **2** inhibited the phosphorylation of Smad2/3 and suppressed the EMT cell marker proteins (N-cadherin, β-catenin, and vimentin), as well as the related messenger ribonucleic acid expression (N-cadherin, matrix metalloproteinase-9, Snail, and vimentin), while compound **1** did not suppress Smad2/3 phosphorylation and the expression of EMT cell markers. Therefore, compound **2** could be a potential candidate for antimetastatic agent development, because it suppresses TGF-β-induced EMT.

## 1. Introduction

Cancer metastasis refers to the migration of cancer cells from the primary tumor site to distant locations through the blood/lymphatic vessels and accounts for 90% of cancer deaths worldwide [1,2,3]. Although metastasis has not yet been identified as a definite mechanism, several transcription factors, such as Snail and β-catenin, play important roles in cancer cell metastasis [4,5]. The process of epithelial cells transforming into cells with metastatic and invasive capacities is referred to as epithelial–mesenchymal transition (EMT), and it is considered a basic phenomenon of tissue and organ formation during embryonic development in vertebrates and invertebrates. This phenomenon has also been observed in the human wound-healing process, and EMT is closely related to the formation and progression of cancer cells [6,7]. It is characterized by the loss of epithelial cells, including the loss of polarity/cell–cell contact and breakdown of basement membranes, which are converted into mesenchymal cells with mobility. The strong expression of EMT-induced transcription factors, such as Snail and β-catenin, in cancer cells is generally known to result in the loss of E-cadherin expression and the induction of N-cadherin and vimentin [4,8].

Marine natural products are prolific sources of bioactive secondary metabolites with novel structures [9]. In particular, several marine natural products are known as potent cytotoxins [10,11]. Some of them have been used as anticancer agents, while some have provided pharmacophores for anticancer drug discovery [10]. Currently, many chemicals inspired by marine natural products are used in the drug development pipeline [12]. However, only a few studies on the EMT-modulating properties of marine natural products or their derivatives have been reported: eribulin mesylate (a Food and Drug Administration-approved synthetic derivative of marine sponge metabolites) [13], halichondramide (marine sponge metabolites) [14], manzamine A (β-carboline alkaloid of a marine sponge) [15], dibromotyrosine derivatives (marine sponge) [16], flaccidoxide-13-acetate (a membrane diterpene of soft coral) [17], and dieckol (a brown algal metabolite) [18]. Recently, it has been suggested that a large portion of marine natural products originate from marine microorganisms, which are regarded as the most promising source of bioactive natural products [19]. More recently, marine-derived microbial natural products with EMT-suppressing activity have been reported. Biemamides [20] and actinomycin V [21] are EMT modulators produced by marine *Streptomyces* sp. Pentabromopseudilin produced by *Pseudomonas* sp. [22] and androsamide produced by *Norcadiopsis* sp. [23] have been reported as EMT inhibitors.

In this regard, we investigated the EMT-suppressing potential of a range of marine microbial culture broth extracts. The ethyl acetate (EtOAc) extract of the culture broth of *Aquimarina* sp. MC085 was one of the successful EMT-suppressing extracts. The bioactivity-guided purification of the extract resulted in the isolation of a new natural product, caprolactin C (**1**). To compare the bioactivity by chirality, the enantiomer of **1**, *ent*-caprolactin C (**2**), was synthesized. Compounds **1** and **2** inhibited the invasion of A549 human lung cancer cells and γ-irradiation (IR)-induced migration of A549 cancer cells. Further mechanistic study on the effect of compound **2** on A549 cancer cells revealed that a treatment with **2** inhibited the transforming growth factor-β (TGF-β)-induced EMT in A549 human lung cancer cells by modulating Smad-dependent signaling.

## 2. Results

**Figure 1:**
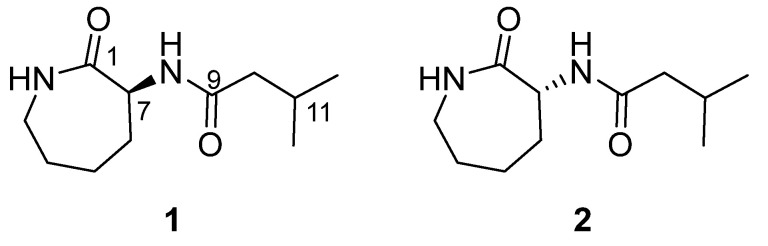
Structures of caprolactin C (**1**, a natural product) and *ent*-caprolactin C (**2**, a synthetic compound).

A variety of bacterial strains were isolated from a marine biomass collected in Kosrae, the Federated States of Micronesia. The EtOAc extracts of the microbial culture (2 L) were prepared, and their invasion and γ-IR-induced migration inhibitory activities, as well as EMT-suppressing activities, were tested on A549 human lung cancer cells. The EtOAc extract of the *Aquimarina* sp. MC085 (GenBank accession no. MG016025) was found to possess excellent invasion and γ-IR-induced migration inhibitory, as well as EMT-suppressing, properties. Bioactivity-guided fractionation of the crude extract of *Aquimarina* sp. MC085 resulted in the discovery of a new A549 cell invasion and a γ-IR-induced migration-inhibiting natural product, caprolactin C (**1**), and its synthetic enantiomer *ent*-caprolactin C (**2**) with EMT-suppressing properties (Figure 1).

### 2.1. Structure Elucidation of Compound ***1***

Compound **1** was obtained as a white powder, [α]_D_^22^ + 15.61° (*c* 0.26, CH_2_Cl_2_). HR–EI–MS revealed a molecular ion peak at *m/z* 212.1526 ([M]^+^ calcd 212.1519, Figure S1), indicating the molecular formula of C_11_H_20_N_2_O_2_ with three degrees of unsaturation.

The ^1^H NMR spectrum of compound **1** displayed two downfield-shifted broad singlet proton signals (δ_H_ 6.84 ppm and 6.00 ppm), which supported the presence of NH (Table 1 and Figure S2). The ^13^C NMR spectrum of compound **1** displayed 11 carbon signals, including two amide carbonyl carbons (δ_C_ 175.75 ppm and 171.80 ppm) and two N-substituted sp3 carbons (δ_C_ 52.20 ppm and 42.36 ppm), as shown in Table 1 and Figure S3. The absence of additional sp2 carbons and the degree of unsaturation suggest that **1** has a cyclic structure.

**Figure 2:**
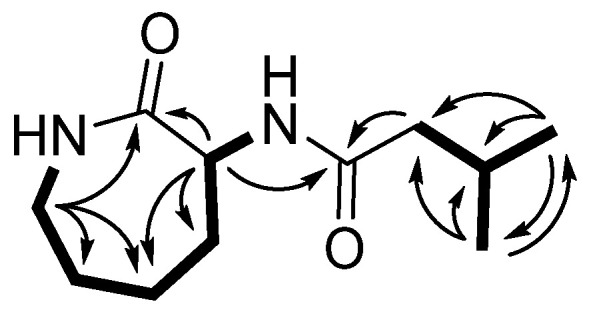
The major HMBC (H→C) and COSY (▬) correlations of **1**.

The planar structure of **1** was determined by two-dimensional NMR spectroscopic data analyses, including correlation spectroscopy (COSY), phase-sensitive heteronuclear single quantum coherence (HSQC), and heteronuclear multiple bond correlation (HMBC) spectra analyses (Figures S4–S6). A series of COSY correlations of **1** revealed two spin systems. The first spin system consisted of four methylene protons (H-3, H-4, H-5, and H-6) and one methine proton (H-7) between two NH protons (H-2 and H-8), as shown in Figure 2. In addition, two doublet methyl protons (H-12 and H-13) exhibited an intense COSY correlation with a methine proton at δ_H_ 2.11 ppm (H-11), which also exhibited a COSY correlation with a methylene proton at δ_H_ 2.09 ppm (H-10). These two discrete spin systems were also confirmed by HMBC correlations from H-3 to C-4/C-5, from H-7 to C-5/C-6, and from H-12/H-13 to C-10 and C-11. In addition, the two major HMBC correlations from H-7/H-10 to C-9 suggested that there was an amide bond connection between the two spin systems. Furthermore, the HMBC correlations from two methylene protons at δ_H_ 3.30 ppm and 3.24 ppm (H-3) and a methine proton at δ_H_ 4.54 ppm (H-7) to one of the carbonyl carbons at δ_C_ 175.75 ppm (C-1) revealed a caprolactam structure possessing an amide bond between C-3 and C-7.

The absolute configuration of caprolactin C (**1**) was determined by comparing the observed specific rotation data with the reported values of structurally related compounds. It has been shown previously that a mixture of caprolactins A and B are cyclic-l-lysine linked to 7-methyloctanoic acid and 6-methyloctanoic acid, respectively. Caprolactins A and B have been reported to have an *S* configuration at the C-7 position of caprolactam, which was assigned using Marfey’s method and confirmed by synthesis [24]. The sign of the optical rotation of compound **1** ([α]_D_^22^ + 15.61°, *c* 0.26, CH_2_Cl_2_) matched that of a mixture of caprolactins A and B ([α]_D_^22^ + 5.4°, *c* 1.03, CH_2_Cl_2_), and the absolute configuration of C-7 on **1** was speculated as *S* [24].

### 2.2. Synthesis of Caprolactin C (***1***) and Ent-Caprolactin C (***2***)

Through the acylation of the racemic mixture of d/l-α-amino-ε-caprolactam, **1** and its enantiomer **2** were synthesized. The resulting reaction products were purified into optically pure compounds **1** and **2** using a chiral high-performance liquid chromatography (HPLC) column. The structures of synthetic compounds **1** and **2** were confirmed by comparing the spectroscopic data, including the one-dimensional NMR spectra, LR–ESI–MS spectra, and specific rotation values (synthetic **1**: [α]_D_^25^ + 21.29 and **2**: [α]_D_^25^ − 46.76).

### 2.3. Effects of Caprolactin C (***1***) and Ent-Caprolactin C (***2***) on the Invasion and γ-IR-Induced Migration of A549 Cells

**Figure 3:**
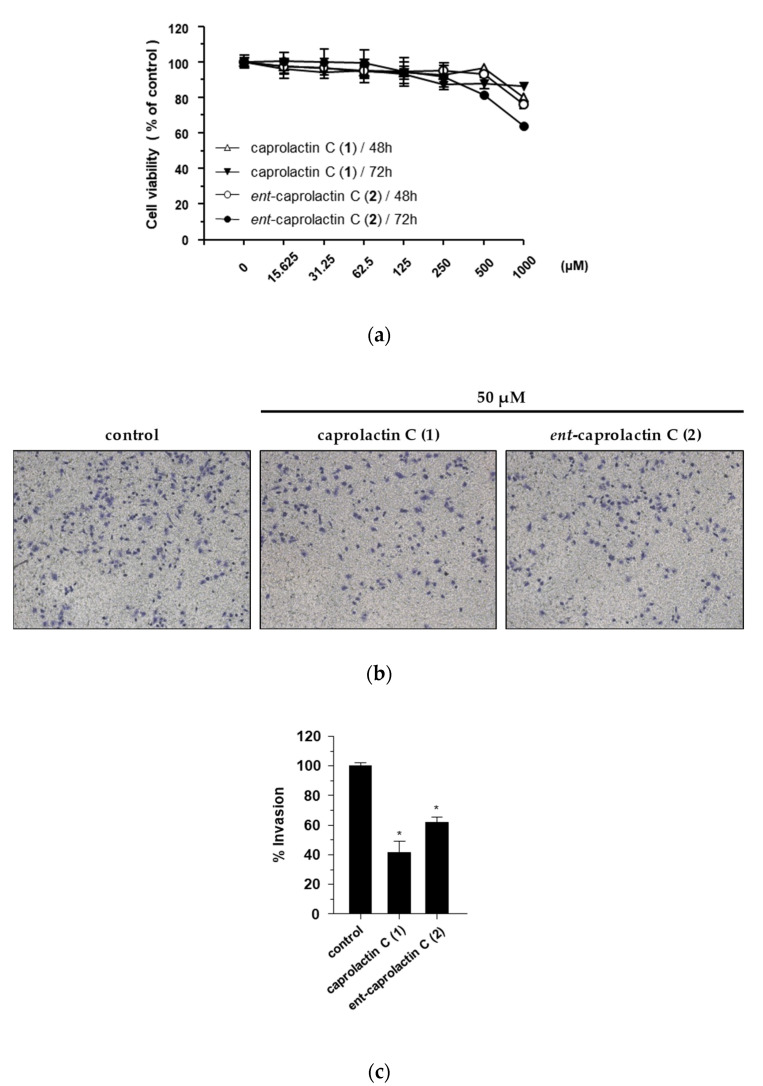
Inhibitory effects of compounds **1** and **2** on the invasion of A549 cells. (**a**) Effects of compounds **1** and **2** on A549 cell viability. (**b**) Effects of compounds **1** and **2** on cell invasion. (**c**) Quantified graph showing the effects of compounds **1** and **2** on cell invasion. Cell viability was estimated using the CCK-8 assay after the treatment with the indicated compounds (15.625–1000 μM) for 48 h and 72 h. Cell invasion was assessed using Matrigel-coated Transwell chambers in the A549 cells treated with 50 μM of compounds **1** and **2**, separately. Quantification of the invasion was displayed as the percentage of invasion representing the number of cells per field compared with the control. The data are presented as the mean ± SD (*n* = 3), * p < 0.05.

The effect of compounds **1** and **2** on the viability of the A549 cells was measured at concentrations of up to 1 mM. Both compounds showed over 90% cell viability at concentrations up to 125 μM (Figure 3a). A549 cell invasion was tested at a concentration of 50 μM, showing over 90% cell viability. The invasion of A549 cells was significantly suppressed by the treatment with **1** and **2**. Compared to the loading control, **1** exhibited 42.61% ± 19.00% A549 cell invasion, while **2** exhibited 62.02% ± 8.39% A549 cell invasion.

**Figure 4:**
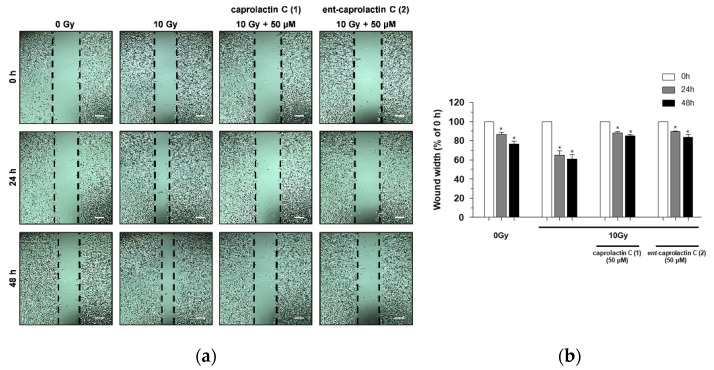
The inhibition of γ-IR-induced A549 cell migration by **1** and **2**. (**a**) A wound-healing assay performed to examine the effects of the indicated compounds (50 μM) on the γ-IR-induced migration of the A549 cells. Scale bar, 100 μm. (**b**) Quantification of the wound width. The relative wound width was calculated as the ratio of the remaining wound width at a specified time point to the original wound width created at 0 h. The data are presented as the mean ± SD (*n* = 3), * *p* < 0.005.

It has been reported that the migration and invasion of A549 cells is promoted by sublethal doses of γ-IR [25,26]. To investigate the inhibitory effects of compounds **1** and **2** on γ-IR-induced A549 cell migration, a wound-healing assay was performed. As shown in Figure 4, caprolactin C (**1**) and *ent*-caprolactin C (**2**) decreased the cover rate of the scratch width, which was confirmed by the quantification of the cell migration. Compounds **1** and **2** inhibited γ-IR-induced A549 cell migration at noncytotoxic concentrations.

### 2.4. Effects of Caprolactin C (***1***) and Ent-Caprolactin C (***2***) on the Viability of the A549 Cells

**Figure 5:**
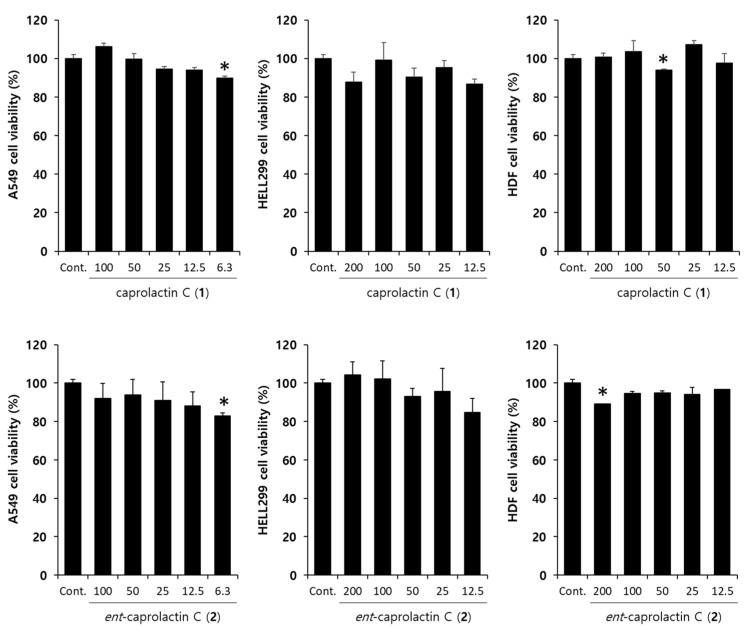
Effect of caprolactin C (**1**) and *ent*-caprolactin C (**2**) on the viability of the A549, HELL299, and human dermal fibroblast (HDF) cells. The cells were plated at a density of 1 × 10^4^ cells/well in a 96-well plate and subsequently treated with **1** and **2** at the indicated concentrations for 48 h. Cell viability was measured by the MTT assay, as described in Section 4. Data are presented as the mean ± SD of three independent experiments. * *p* < 0.01 versus the control group.

To investigate the effects of **1** and **2** on TGF-β-induced EMT, we assessed the toxicity of **1** and **2** in A549 cells. The cells were pretreated with different concentrations (100, 50, 25, 12.5, and 6.5 µM) of **1** and **2** for 6 h and subsequently stimulated with TGF-β for 42 h. As shown in Figure 5, the viability of the A549 cells was above 80% regardless of the concentration compared to that of the loading control. Compounds **1** and **2** had no influence on the cell proliferation or toxicity in A549 cells at the indicated concentrations.

### 2.5. Effects of Caprolactin C (***1***) and Ent-Caprolactin C (***2***) on TGF-β-Induced EMT of A549 Cells

**Figure 6:**
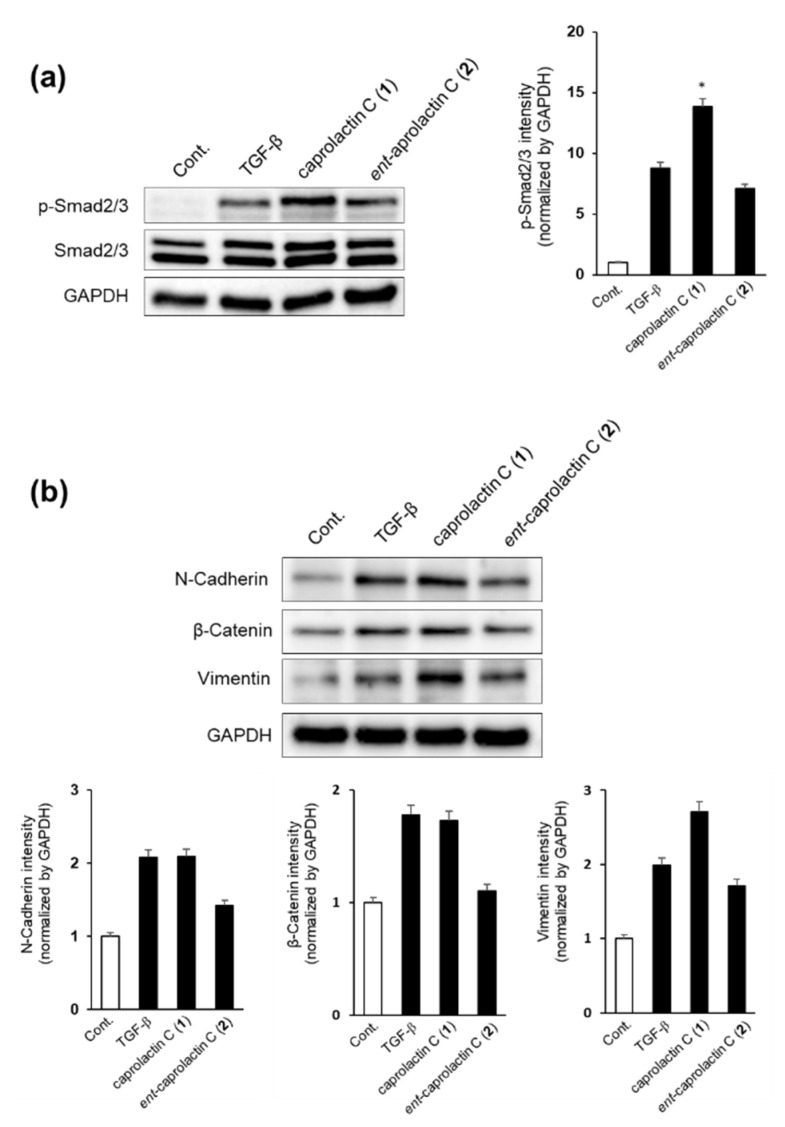
Effect of caprolactin C (**1**) and *ent*-caprolactin C (**2**) on the TGF-β-induced EMT of the A549 cells. (**a**,**b**) The A549 cells were plated at a density of 3 × 10^5^ cells/well in a 6-well plate and subsequently treated with each compound (50 μM) for 6 h. Following the treatment, the cells were stimulated with TGF-β (5 ng/mL) for another 42 h. Whole-cell lysates were immunoblotted with specific antibodies on the left side of each panel. GAPDH served as an internal loading control. The bar chart displays the intensity of N-cadherin, β-catenin, and vimentin after normalization by GAPDH using ImageJ software. Data are presented as the mean ± SD of three independent experiments. * *p* < 0.01 versus the TGF-β treatment group.

We first investigated whether caprolactin C (**1**) or *ent*-caprolactin C (**2**) could inhibit the phosphorylation of Smad2/3 by TGF-β. As shown in Figure 6a, the TGF-β-induced phosphorylation of Smad2/3 was inhibited by the treatment with **2**, but **1** increased the Smad2/3 phosphorylation in A549 cells. We quantified the intensity of the immunoblot bands using ImageJ software. As shown in Figure 6a, **2** slightly inhibited TGF-β-induced p-Smad2/3, while **1** increased TGF-β-induced p-Smad2/3. Next, we examined the expression of mesenchymal cell marker proteins, such as N-cadherin, β-catenin, and vimentin, after the A549 cells were treated with **1** or **2**.

As shown in Figure 6b, the expressions of mesenchymal cell marker proteins such as N-cadherin and β-catenin were decreased significantly by the treatment with *ent*-caprolactin C (**2**), while vimentin expression was slightly decreased by the treatment with **2**. These results indicate that **2** can suppress EMT by regulating Smad2/3 phosphorylation in A549 cells. However, the treatment with **1** increased the expression of vimentin.

### 2.6. Effects of Caprolactin C (***1***) and Ent-Caprolactin C (***2***) on TGF-β-Induced mRNA Expression Levels of N-Cadherin, MMP-9, Snail, and Vimentin in A549 Cells

**Figure 7:**
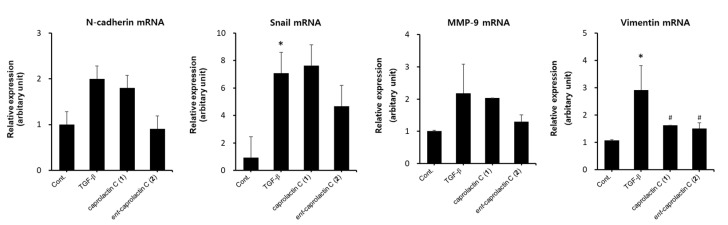
Effect of caprolactin C (**1**) or *ent*-caprolactin C (**2**) on the TGF-β-induced mRNA expression levels of N-cadherin, Snail, MMP-9, and vimentin in the A549 cells. The A549 cells were plated at a density of 3 × 10^5^ cells/well in a 6-well plate and subsequently treated with compounds **1** and **2** (50 μM each) for 6 h. Following the treatment, the cells were stimulated with TGF-β (5 ng/mL) for another 42 h. The expression levels of N-cadherin, Snail, MMP-9, and vimentin mRNA were quantified using qRT-PCR, as described in Section 4. Data are presented as the mean ± SD of three independent experiments. * *p* < 0.01 versus the control group; # *p* < 0.05 versus the TGF-β-treated group.

Next, we investigated whether *ent*-caprolactin C (**2**) inhibited EMT at the mRNA expression level in A549 cells. As shown in Figure 7, the mRNA expression levels of the TGF-β-induced mesenchymal markers, such as N-cadherin, MMP-9, Snail, and vimentin, were inhibited by **2**. The treatment with caprolactin C (**1**) did not affect the mRNA expression levels of N-cadherin, MMP-9, and Snail in A549 cells. However, vimentin mRNA expression was suppressed by both compounds **1** and **2**. Thus, further investigations, such as checking the mRNA expression levels at different time courses in A549 cells, are required. Collectively, these results indicate that **2** is an effective agent for the suppression of EMT.

## 3. Discussion

Cancer metastasis occurs when cancer cells with mesenchymal characteristics (mesenchymal cells) leave their primary sites and enter blood vessels. Therefore, suppressing the mesenchymal characteristics of epithelial cells is important for the development of anticancer agents. Recently, the TGF-β-induced EMT program has been recognized as a promising target for cancer metastasis chemotherapy.

TGF-β is crucial in embryonic development, tissue repair, and tissue homeostasis. In the early stages of tumorigenesis, TGF-β functions as a tumor suppressor, while, at later stages, it functions as a tumor promoter, inducing EMT [27,28]. TGF-β signaling is classified into two types: the Smad-dependent and Smad-independent signaling pathways [29]. When TGF-β binds to its receptor, Smad2/3 is phosphorylated. Thereafter, phosphorylated Smad2/3 forms a complex with Smad4 and translocates to the nucleus. Activated transcription factors, such as zinc-finger E-box-binding homeobox, Snail, and Slug, can induce EMT. Smad-independent signaling can also induce EMT mediated by phosphatidylinositol 3 kinase–protein kinase B, mitogen-activated protein kinase, and MMPs [29].

In this study, we isolated **1** from the culture broth of *Aquimarina* sp. MC085, suppressing A549 cell invasion. Based on the spectroscopic data analysis, its chemical structure was identified as α-amino-ε-caprolactam linked to 3-methyl butanoic acid. Furthermore, the synthesis of **1** produced an enantiomeric pair, **2**.

Compounds **1** and **2** inhibited the invasion and γ-IR-induced migration of A549 cells. In the mechanism study, **2** was found to inhibit the phosphorylation of Smad2/3, which is an initial step in the TGF-β signaling pathway. In addition, the treatment with **2** suppressed the expression of mesenchymal cell markers, such as N-cadherin, β-catenin, and vimentin, as well as invasion markers, such as MMP-9. Therefore, *ent*-caprolactin C (**2**) could be a promising candidate for the development of antimetastatic agents by suppressing the TGF-β-induced EMT pathway.

In contrast, **1** did not inhibit TGF-β-induced mesenchymal markers in A549 cells but inhibited A549 cell invasion and γ-IR-induced cell migration. These results suggest that **1** has another mechanism of action that inhibits the invasion and γ-IR-induced migration of A549 cells.

Compounds **1** and **2** were analyzed to have different mechanisms of action in the aspects of bioactivity that support the importance of absolute configuration in the activity of natural products.

Although the anticancer effects of marine natural products are of significant interest, promising preclinical studies are not generally translated into clinical outcomes. Given the complex processes of initial target identification, hit identification, lead optimization, and the selection of a candidate molecule for clinical development [30,31], further research is needed to comprehensively elucidate the anticancer effects of compounds **1** and **2**. In addition, the lack of knowledge on the stability, bioavailability, and metabolism of these compounds is a limitation of the current study that needs to be clarified in future animal experiments.

## 4. Materials and Methods

### 4.1. General Experimental Procedures

Optical rotation was measured using a Jasco DIP-1000 polarimeter (Tokyo, Japan). Nuclear magnetic resonance (NMR) spectra were recorded using a 250-MHz Bruker NMR spectrometer (DMX 250) and 600-MHz Varian NMR spectrometer (VNS-600, Palo Alto, CA, USA) at the Core Research Support Center for Natural Products and Medical Materials (CRCNM). High-resolution electron ionization mass spectrometry (HR–EI–MS) was performed using a JMS-700 instrument (JEOL, Tokyo, Japan) coupled to a 6890 Series gas chromatography MS (GC–MS) system (Agilent Technologies, Santa Clara, CA, USA). Low-resolution electrospray ionization MS (LR–ESI–MS) was performed using an Agilent 6120 single quadrupole mass spectrometer (Agilent Technologies, Santa Clara, CA, USA) with a reversed-phase C18 column [Phenomenex Luna 3 μm C18(2) 100 Å, new column, 150 × 4.6 mm (Phenomenex, Torrance, CA, USA)]. Isolation of the compounds was carried out using a Waters 1525 binary HPLC pump with a Waters 996 photodiode array (PDA) (Waters Corp., Milford, MA, USA) with a reversed-phase HPLC [RS Tech, Hector-M 5 μm C18, 250 × 4.6 mm (RStech Corp., Cheongju, Republic of Korea)] and an Alltech 301 HPLC pump (Alltech, Lexington, KY, USA) with a Waters 2487 dual wavelength absorbance detector and a chiral HPLC column [CHIRALPAK AD-H 5 μm, 250 × 4.6 mm (Daicel, Osaka, Japan)].

### 4.2. Isolation and Identification of Bacterial Strain

The MC085 bacterial strain was isolated from the consortium of unidentified marine tunicates (15C047, the voucher is deposited at Yeungnam University) collected in Kosrae, the Federated States of Micronesia. The squashed marine biomass was diluted in sterilized seawater and spread on a SYP agar medium (soluble starch, 10 g; yeast extract, 4 g; peptone, 2 g; agar, 16 g; and sterilized seawater, 1 L) plate. The plate was incubated at 25 °C for a week, and a white colony was selected and subcultured on another SYP agar medium plate. The MC085 bacterial strain (GenBank accession No. MG016025) was identified as an *Aquimarina* sp. (98.32% similarity in the 16S ribosomal ribonucleic acid (rRNA) sequence to *Aquimarina salinaria* strain antisso-27), and the strain was stored at the College of Pharmacy, Yeungnam University.

### 4.3. Extraction and Isolation of Compound ***1***

The bacterial strain *Aquimarina* sp. MC085 was placed in 2 L of SYP seawater liquid medium for 7 days at 25 °C with shaking at 150 rpm. After cultivation, the culture broth was extracted twice with EtOAc, and the combined extract was evaporated under reduced pressure. The dried crude extract (223.2 mg) was separated into seven fractions by silica gel small column chromatography using a step-gradient eluent mixture of dichloromethane and methanol (from 100:0 to 0:100). Fraction 5 (42.1 mg, eluted with CH_2_Cl_2_:MeOH = 10:1) was subjected to reversed-phase HPLC (Hector-M 5 μm C18, 250 × 4.6 mm) with a stepwise gradient of MeOH:H_2_O = 25:75 → 50:50 (210 nm ultraviolet region) to afford **1** (2.8 mg).

#### Caprolactin C (**1**)

White powder; [α]_D_^25^ + 15.61 (*c* 0.26, CH_2_Cl_2_); ^1^H and ^13^C NMR data, see Table 1 and Supplementary Data; HR–EI–MS [M]^+^ *m/z* 212.1526 (calcd for C_11_H_20_N_2_O_2_^+^, 212.1519).

### 4.4. Synthesis of Caprolactin C (***1***) and Ent-Caprolactin C (***2***)

Compound **1** was synthesized to provide a sufficient material for pharmacological investigations. A racemic mixture of d/l-α-amino-ε-caprolactam (51.28 mg, Tokyo Chemical Industry, Tokyo, Japan) and Na_2_CO_3_ (127.2 mg) in water (5 mL) was added to a solution of isovaleryl chloride (49 µL) in CH_2_Cl_2_ (5 mL) at 25 °C, and the flask was incubated overnight. The organic layer was separated, and the residual aqueous phase was extracted twice using CH_2_Cl_2_ (5 mL). The combined organic layers were dried under reduced pressure. The residue was subjected to HPLC using a chiral column (CHIRALPAK AD-H, 5 μm, 250 × 4.6 mm) to separate the chiroptically pure compound **1** and its enantiomeric pair. Isocratic elution of the mixed solvent (*n*-hexane:isopropyl alcohol = 90:10) resulted in the isolation of **1** (retention time of 13.44 min, 27.2 mg) and **2** (retention time of 8.75 min, 22.9 mg).

#### 4.4.1. Synthetic Caprolactin C (**1**)

White powder; [α]_D_^25^ + 21.29 (*c* 0.44, CH_2_Cl_2_); ^1^H and ^13^C NMR data, see Supplementary Data; HR–EI–MS [M]^+^ *m/z* 212.1526 (calcd for C_11_H_20_N_2_O_2_^+^, 212.1519).

#### 4.4.2. Synthetic Ent-Caprolactin C (**2**)

White powder; [α]_D_^25^ − 46.76 (*c* 0.26, CH_2_Cl_2_); ^1^H and ^13^C NMR data, see Supplementary Data; HR–EI–MS [M]^+^ *m/z* 212.1526 (calcd for C_11_H_20_N_2_O_2_^+^, 212.1519).

### 4.5. Biological Assays

#### 4.5.1. Invasion Assay

The cell invasion potential of the compounds was assessed using a Transwell assay, as described previously [25]. In summary, the cells in a serum-free medium were seeded (1.5 × 10^4^ cells) onto the upper surfaces of Matrigel-coated Transwell chambers (BD Biosciences, Franklin Lakes, NJ, USA), and 50 μM of caprolactin C (**1**) and *ent*-caprolactin C (**2**) were added, separately. The lower compartments of the chambers were filled with the medium supplemented with 10% fetal bovine serum (FBS). After 16 h of incubation, the cells that invaded the lower surface of the filter were fixed with 70% ethanol for 10 min and stained with 0.2% crystal violet for 10 min. The invaded cells were counted under an Eclipse TE2000-U microscope (Nikon).

#### 4.5.2. γ-IR-Induced Migration Assay (Wound-Healing Assay)

A549 cells were seeded in a 24-well plate (3.5 × 10^5^ cells/well) and incubated overnight. To generate a wound area, the cells were scratched with a 200-μL pipette tip and exposed to 10 Gy of γ-IR using a ^137^Cs γ-ray source (Atomic Energy of Canada, Mississauga, ON, Canada) at a dose rate of 3 Gy/min. The irradiated cells were incubated for 24 h with 50 μM of caprolactin C (**1**) and *ent*-caprolactin C (**2**) each. The images were analyzed at 0 h and 24 h using an AE31 microscope (Motic, Hong Kong).

#### 4.5.3. Antibodies and Reagents

Antibodies against N-cadherin (D4R1H), vimentin (D21H3), β-catenin (D10A8), Smad2/3 (D7G7), and glyceraldehyde 3-phosphate dehydrogenase (GAPDH) (14C10) were purchased from Cell Signaling Technology (Danvers, MA, USA). Phospho-specific antibodies against Smad2/3 (Ser465/Ser467) were purchased from Cell Signaling Technology. The RPMI 1640 medium was obtained from GE Healthcare Life Sciences (Piscataway, NJ, USA), and FBS was purchased from ATCC (Manassas, VA, USA). Recombinant human TGF-β was purchased from Peprotech (Rocky Hill, NJ, USA).

#### 4.5.4. Cell Culture

A549 and HEL299 cells were purchased from Korean Cell Line Bank (Seoul, Republic of Korea), and HDF was obtained from ATCC (Manassas, VA, USA). A549 and HEL299 were maintained in an RPMI 1640 medium, and HDF was maintained in Dulbecco’s modified Eagle’s medium. All media for the cells were supplemented with 10% FBS, 2-mM glutamine, 1-mM sodium pyruvate, 100 U/mL of penicillin, and 100 μg/mL of streptomycin. The cells were incubated at 37 °C in a 5% CO_2_ incubator. The cells were subcultured every three days to maintain the monolayer cells.

#### 4.5.5. Cell Viability Measurement

Human lung adenocarcinoma A549 cells were treated with various concentrations (6.3–100 μM) of caprolactin C (**1**) or *ent*-caprolactin C (**2**) for 48 h. Then, the supernatant was removed by aspiration, and a 10-times diluted Cell Counting Kit-8 (CCK-8) solution was added. The A549 cells were incubated for 1 h, and the absorbance values were measured at 450 nm. The percentage of cell viability was measured by comparing it to that of the loading control group.

#### 4.5.6. Preparation of Cell Lysates for Immunoblotting

The A549 cells were preincubated in a 6-well plate overnight, washed with phosphate-buffered saline (PBS), and incubated with serum-free medium containing 50 μM of **1** and **2**. After 6 h of incubation, the cells were stimulated with TGF-β (5 ng/mL) for another 42 h. Thereafter, the cells were washed twice with cold PBS and lysed with a radioimmunoprecipitation assay buffer (T&I, Seoul, Korea) supplemented with 1-mM dithiothreitol (DTT), 10-mM β-glycerophosphate, 1-mM sodium orthovanadate, 1-mM phenylmethylsulphonyl fluoride (PMSF), and a protease inhibitor cocktail (Roche Diagnostics Corp., Indianapolis, IN, USA). The cell lysate was collected and centrifuged at 13,000 rpm for 20 min at 4 °C. The total protein amount was measured using a bicinchoninic acid assay, and the concentration of each sample was adjusted accordingly. Sodium dodecyl sulfate-polyacrylamide gel electrophoresis, protein transfer, and the membrane development were performed as described in previous reports [32,33]. The protein concentration was determined using the Pierce™ BCA Protein Assay Kit (Pierce, Rockford, IL, USA). The proteins were separated using sodium dodecyl sulfate-polyacrylamide gel electrophoresis. Then, the proteins were transferred to a polyvinylidene difluoride membrane (Merck Millipore, Darmstadt, Germany) and blocked with 5% skimmed milk. The membranes were incubated at room temperature with diluted primary antibodies, including p-Smad2/3 (1:1000, #8828, Cell Signaling Technology, Danvers, MA, USA), Smad2/3 (1:1000, #3102, Cell Signaling Technology), N-cadherin (1:1000, #14215, Cell Signaling Technology), β-catenin (1:1000, #9562, Cell Signaling Technology), vimentin (1:1000, #3932, Cell Signaling Technology), and GAPDH (1:5000, #2118, Cell Signaling Technology), for 4 h. The membrane was washed and incubated at room temperature with rabbit IgG secondary antibody (1:5000, #7074, Cell Signaling Technology) or mouse IgG secondary antibody (1:5000, #7076, Cell Signaling Technology) for 1 h. The protein signals were measured using SuperSignal^®^ West Femto Maximum Sensitivity Chemiluminescent Substrate (Pierce) and the Fusion Solo Chemiluminescence System (PEQLAB Biotechnologie GmbH, Erlangen, Germany). The protein expression was normalized to that of the GAPDH reference protein. The analysis was performed using a Fusion Solo Chemiluminescence System (PEQLAB Biotechnologie GmbH, Erlangen, Germany). The relative protein expression was calculated compared to an untreated group using ImageJ software (National Institutes of Health, Bethesda, MD, USA). ImageJ software (National Institutes of Health, Bethesda, MD, USA) was used to quantify the relative protein expression in comparison with that of the loading controls.

#### 4.5.7. Quantitative Reverse Transcription-Polymerase Chain Reaction (qRT-PCR)

The total RNA was isolated from the cells using a RNeasy Mini Kit (Qiagen, Valencia, CA, USA) according to the manufacturer’s protocols. For qRT-PCR, 1 µg of total RNA was converted into a complementary deoxyribonucleic acid (cDNA) using a RevertAid First Strand cDNA Synthesis Kit (Fermentas, MA, USA). To amplify the cDNA, the reverse-transcribed cDNA was subjected to 40 cycles of qRT-PCR using the PowerUp SYBR Green PCR Master Mix (Thermo Fisher Scientific, Waltham, MA, USA) with sense and antisense primers at 200 nM. The primers were 5′-GAGAACTTTGCCGTTGAAGC-3′ and 5′-GCTTTCTGTAGGTGGCAATC-3′ for human vimentin, 5′-CGGAGTGAGTTGAACCAG-3′ and 5′-GTCCCAGTGGGGATTTAC-3′ for human matrix metalloproteinase-9 (MMP-9), and 5′-GAAGGTGAAGGTCGGAGTC-3′ and 5′-GAAGATGGTGATGGGATTT-3′ for GAPDH. The vimentin and MMP-9 messenger RNA (mRNA) expression levels were normalized to the corresponding expression levels of GAPDH. The relative expression levels were determined via qRT-PCR using the Quant 3 Real-Time PCR system (Thermo Fisher Scientific, Waltham, MA, USA).

#### 4.5.8. Statistical Analysis

The results are expressed as the mean ± SD of triplicate experiments. The results were statistically analyzed using the Mann–Whitney *U* test using Prism 8 (GraphPad Software, San Diego, CA, USA).

## Figures and Tables

**Table 1:** ^1^H and ^13^C NMR spectroscopic data of compound **1** with HMBC correlations.

Position	Caprolacin C (1)		
	*δ*_C_^1^, Mult.	*δ*_H_^2^, Mult. (*J* in Hz)	HMBC (Carbon No.)
1	175.75, C		
2		6.00, br s	
3a	42.36, CH_2_	3.30, m	1, 4, 5
3b		3.24, m	1, 4, 5
4a	29.09, CH_2_	1.84 ^3^, m	3, 5, 6
4b		1.39, m	3, 6
5a	28.08, CH_2_	1.99, m	3, 4, 7, 6
5b		1.84 ^3^, m	3, 4, 6, 7
6a	31.90, CH_2_	2.09 ^3^, m	4, 5
6b		1.47, m	4, 5, 7
7	52.20, CH	4.54, ddd (11.34, 5.94, 1.98)	1, 5, 6, 9
8		6.84, br s	
9	171.80, C		
10	46.09, CH_2_	2.09 ^3^, m	9, 11, 12, 13
11	26.33, CH	2.11, m	9, 10, 12, 13
12	22.67, CH_3_	0.96, d (6.01)	10, 11, 13
13	22.51, CH_3_	0.95, d (6.20)	10, 11, 12

^1 13^C NMR spectrum was recorded at 150 MHz. ^2 1^H NMR spectrum was recorded at 600 MHz. ^3^ Overlapped.

## Data Availability

The article contains all the data produced in this study.

## Supplementary Materials

The following are available online at https://www.mdpi.com/article/10.3390/md19080465/s1: Figure S1: HR-EI-MS data of **1**. Figure S2: ^1^H NMR spectrum (600 MHz) of **1** in CDCl_3_. Figure S3: ^13^C NMR spectrum (150 MHz) of **1** in CDCl_3_. Figure S4: COSY spectrum of **1** in CDCl_3_. Figure S5: Phase-sensitive HSQC spectrum of **1** in CDCl_3_. Figure S6: HMBC spectrum of **1** in CDCl_3_. Figure S7: Chiral separation of synthetic compounds **1** (synthetic caprolactam 2) and **2** (synthetic caprolactam 1). Figure S8: ^1^H NMR spectrum (250 MHz) of **2** (a synthetic caprolactam 1) in CDCl_3_. Figure S9: ^13^C NMR spectrum (63 MHz) of **2** in CDCl_3_. Figure S10: Comparison of ^1^H NMR spectra of **1** (natural), **1** (synthetic), and **2** (synthetic).

## References

[1] Chambers A.F., Groom A.C., MacDonald I.C. (2002). Dissemination and growth of cancer cells in metastatic sites. Nat. Rev. Cancer.

[2] Chaffer C.L., Weinberg R.A. (2011). A perspective on cancer cell metastasis. Science.

[3] Fidler I.J. (2003). The pathogenesis of cancer metastasis: The ’seed and soil’ hypothesis revisited. Nat. Rev. Cancer.

[4] Mahmood M.Q., Walters E.H., Shukla S.D., Weston S., Muller H.K., Ward C., Sohal S.S. (2017). β-catenin, twist and snail: Transcriptional regulation of EMT in smokers and COPD, and relation to airflow obstruction. Sci. Rep..

[5] Wang Y., Shi J., Chai K., Ying X., Zhou B.P. (2013). The role of snail in EMT and tumorigenesis. Curr. Cancer Drug Targets.

[6] Nieto M.A. (2009). Epithelial–mesenchymal transitions in development and disease: Old views and new perspectives. Int. J. Dev. Biol..

[7] Stone R.C., Pastar I., Ojeh N., Chen V., Liu S., Garzon K.I., Tomic-Canic M. (2016). Epithelial-mesenchymal transition in tissue repair and fibrosis. Cell Tissue Res..

[8] Lamouille S., Xu J., Derynck R. (2014). Molecular mechanisms of epithelial–mesenchymal transition. Nat. Rev. Mol. Cell Biol..

[9] Carroll A.R., Copp B.R., Davis R.A., Keyzers R.A., Princep M.R. (2020). Marine natural products. Nat. Prod. Rep..

[10] Newman D.J., Cragg G.M. (2020). Natural products as sources of new drugs over the nearly four decades from 01/1981 to 09/2019. J. Nat. Prod..

[11] Nigam M., Suleria H.A.R., Farzaei M.H., Mishra A.P. (2019). Marine anticancer drugs and their relevant targets: A treasure from the ocean. DARU J. Pharm. Sci..

[12] Mayer A.M.S., Glaser K.B., Cuevas C., Jacobs R.S., Kem W., Little R.D., McIntosh J.M., Newman D.J., Potts B.C., Shuster D.E. (2010). The odyssey of marine pharmaceuticals: A current pipeline perspective. Trends Pharmacol. Sci..

[13] Dybdal-Hargreaves N.F., Risinger A.L., Mooberry S.L. (2015). Eribulin mesylate: Mechanism of action of a unique microtubule targeting agent. Clin. Cancer Res..

[14] Shin Y., Kim G.D., Jeon J.-E., Shin J., Lee S.K. (2013). Antimetastatic effect of halichondramide, a trisoxazole macrolide from the marine sponge *Chondrosia corticata*, on human prostate cancer cells via modulation of epithelial-to-mesenchymal transition. Mar. Drugs.

[15] Lin L.-C., Kuo T.-T., Chang H.-Y., Liu W.-S., Hsia S.-M., Huang T.-C. (2018). Manzamine A exerts anticancer activity against human colorectal cancer cells. Mar. Drugs.

[16] Chen C.L., Kao Y.C., Yang P.H., Sung P.J., Wen Z.H., Chen J.J., Huang Y.B., Chen P.Y. (2016). A small dibromotyrosine derivative purified from *Pseudoceratina* sp. suppresses TGF-β responsiveness by inhibiting TGF-β type I receptor serine/threonine kinase activity. J. Cell Biochem..

[17] Wu Y.-J., Wei W.-C., Dai G.-F., Su J.-H., Tseng Y.-H., Tsai T.-C. (2020). Exploring the mechanism of flaccidoxide-13-acetate in suppressing cell metastasis of hepatocellular carcinoma. Mar. Drugs.

[18] Jeong S.H., Jeon Y.J., Park S.J. (2016). Inhibitory effects of dieckol on hypoxia-induced epithelial-mesenchymal transition of HT29 human colorectal cancer cells. Mol. Med. Rep..

[19] Gerwick W.H., Moore B.S. (2012). Lessons from the past and charting the future of marine natural products drug discovery and chemical biology. Chem. Biol..

[20] Zhang F., Braun D.R., Ananiev G.E., Hoffmann F.M., Tsai I.W., Rajski S.R., Bugni T.S. (2018). Biemamides A-E, inhibitors of the TGF-β pathway that block the epithelial to mesenchymal transition. Org. Lett..

[21] Lin S., Zhang C., Liu F., Ma J., Jia F., Han Z., Xie W., Li X. (2019). Actinomycin V inhibits migration and invasion via suppressing snail/slug-mediated epithelial-mesenchymal transition progression in human breast cancer MDA-MB-231 cells in vitro. Mar. Drugs.

[22] Shih-Wei W., Chih-Ling C., Kao Y.C., Martin R., Knölker H.J., Shiao M.S., Chen C.L. (2018). Pentabromopseudilin: A myosin V inhibitor suppresses TGF-β activity by recruiting the type II TGF-β receptor to lysosomal degradation. J. Enzyme Inhib. Med. Chem..

[23] Lee J., Gamage C.D.B., Kim G.J., Hillman P.F., Lee C., Lee E.Y., Choi H., Kim H., Nam S.J., Fenical W. (2020). Androsamide, a cyclic tetrapeptide from a marine *Nocardiopsis* sp., suppresses motility of colorectal cancer cells. J. Nat. Prod..

[24] Davidson B.S., Schumacher R.W. (1993). Isolation and synthesis of caprolactins A and B, new caprolactams from a marine bacterium. Tetrahedron.

[25] Han A.R., Lee S., Han S., Lee Y.J., Kim J.B., Seo E.K., Jung C.H. (2020). Triterpenoids from the leaves of *Centella asiatica* inhibit ionizing radiation-induced migration and invasion of human lung cancer cells. Evid. Based Complementary Altern. Med..

[26] Jung C.H., Kim E.M., Song J.Y., Park J.K., Um H.D. (2019). Mitochondrial superoxide dismutase 2 mediates γ-irradiation-induced cancer cell invasion. Exp. Mol. Med..

[27] Derynck R., Akhurst R.J., Balmain A. (2001). TGF-β signaling in tumor suppression and cancer progression. Nat. Genet..

[28] Ikushima H., Miyazono K. (2020). TGFβ signalling: A complex web in cancer progression. Nat. Rev. Cancer.

[29] Liu S., Chen S., Zeng J. (2018). TGF-β signaling: A complex role in tumorigenesis. Mol. Med. Rep..

[30] Hughes J.P., Rees S., Kalindjian S.B., Philpott K.L. (2011). Principles of early drug discovery. Br. J. Pharmacol..

[31] Thomford N.E., Senthebane D.A., Rowe A., Munro D., Seele P., Maroyi A., Dzobo K. (2018). Natural products for drug discovery in the 21st century: Innovations for novel drug discovery. Int. J. Mol. Sci..

[32] Kim H.W., Shin M.S., Lee S.J., Park H.R., Jee H.S., Yoon T.J., Shin K.S. (2019). Signaling pathways associated with macrophage-activating polysaccharides purified from fermented barley. Int. J. Biol. Macromol..

[33] Park J.Y., Shin M.S. (2021). Inhibitory effects of pectic polysaccharide isolated from *Diospyros kaki* leaves on tumor cell angiogenesis via VEGF and MMP-9 regulation. Polymers.

