# Supplementary material for: Inhibition of A549 Lung Cancer Cell Migration and Invasion by Ent-Caprolactin C via the Suppression of Transforming Growth Factor-β-Induced Epithelial—Mesenchymal Transition
Source: Mar Drugs. 2021 Aug 19;19(8):465. doi: 10.3390/md19080465 (PMC8398538; doi:10.3390/md19080465)

Inhibition of A549 Lung Cancer Cell Migration and Invasion by *Ent*-Caprolactin C via the Suppression of Transforming Growth Factor-β-Induced Epithelial–Mesenchymal Transition

So Young Kim [1,†], Myoung-Sook Shin [2,†], Geum Jin Kim [1,3], Hyukbean Kwon [1], Myong Jin Lee [2], Ah-Reum Han [4], Joo-Won Nam [1], Chan-Hun Jung [5], Ki Sung Kang [2,*] and Hyukjae Choi [1,3,*]

[1] College of Pharmacy, Yeungnam University, Gyeongsan-si, Gyeongsangbukdo 38541, Korea; sososo0305@hanmail.net (S.Y.K.); canta87@ynu.ac.kr (G.J.K.); zero9602@gmail.com (H.K.); jwnam@yu.ac.kr (J.-W.N.)

[2] College of Korean Medicine, Gachon University, Seongnam 13120, Korea; ms.shin@gachon.ac.kr (M.-S.S.); myongene@naver.com (M.J.L.)

[3] Research Institute of Cell Culture, Yeungnam University, Gyeongsan-si, Gyeongsangbukdo 38541, Korea

[4] Advanced Radiation Technology Institute, Korea Atomic Energy Research Institute, Jeongeup-si, Jeollabuk-do 56212, Korea; arhan@kaeri.re.kr

[5] Jeonju AgroBio-Materials Institute, Jeonju-si, Jeollabuk-do 54810, Korea; biohun@gmail.com

* Correspondence: kkang@gachon.ac.kr (K.S.K.); h5choi@yu.ac.kr (H.C.); Tel.: +82-31-750-5402 (K.S.K.); Tel.: +82-53-810-2824 (H.C.)

† These authors contributed equally to this work.

Figure S1. HR-EI-MS data of **1**

Figure S2. ^{1}H NMR spectrum (600 MHz) of **1** in $CDCl_3$

Figure S3. ^{13}C NMR spectrum (150 MHz) of **1** in $CDCl_3$

Figure S4. COSY spectrum of **1** in $CDCl_3$

Figure S5. Phase-sensitive HSQC spectrum of **1** in $CDCl_3$

Figure S6. HMBC spectrum of **1** in $CDCl_3$

Figure S7. Chiral separation of synthetic compounds **1** (synthetic caprolactam 2) and **2** (synthetic caprolactam 1)

Figure S8. ^{1}H NMR spectrum (250 MHz) of **2** (a synthetic caprolactam 1) in $CDCl_3$

Figure S9. ^{13}C NMR spectrum (63 MHz) of **2** in $CDCl_3$

Figure S10. Comparison of ^{1}H NMR spectra of **1** (natural), **1** (synthetic) and **2** (synthetic)

Figure S1. HR-EI-MS data of **1**

```
[ Elemental Composition ]                                                   Page: 1
Data   : EI-A407                         Date : 25-Jan-2017 14:08
Sample: MC085-E-5
Note : -
Inlet : Direct                           Ion Mode : EI+
RT : 0.99 min                            Scan#: (50,51)
Elements : C 100/0, H 150/0, N 10/0, O 10/0
Mass Tolerance       : 1000ppm, 3mmu if m/z < 3, 5mmu if m/z > 5
Unsaturation (U.S.) : -0.5 - 50.0

Observed m/z Int%   Err[ppm / mmu]    U.S.  Composition
  212.1526   100.0    -18.4 /  -3.9    7.0  C 16 H 20
                      +13.2 /  +2.8    4.0  C 7 H 16 N 8
                       +6.9 /  +1.5    3.5  C 9 H 18 N 5 O
                       +0.6 /  +0.1    3.0  C 11 H 20 N 2 O 2
```

Figure S2. ^{1}H NMR spectrum (600 MHz) of **1** in $CDCl_3$

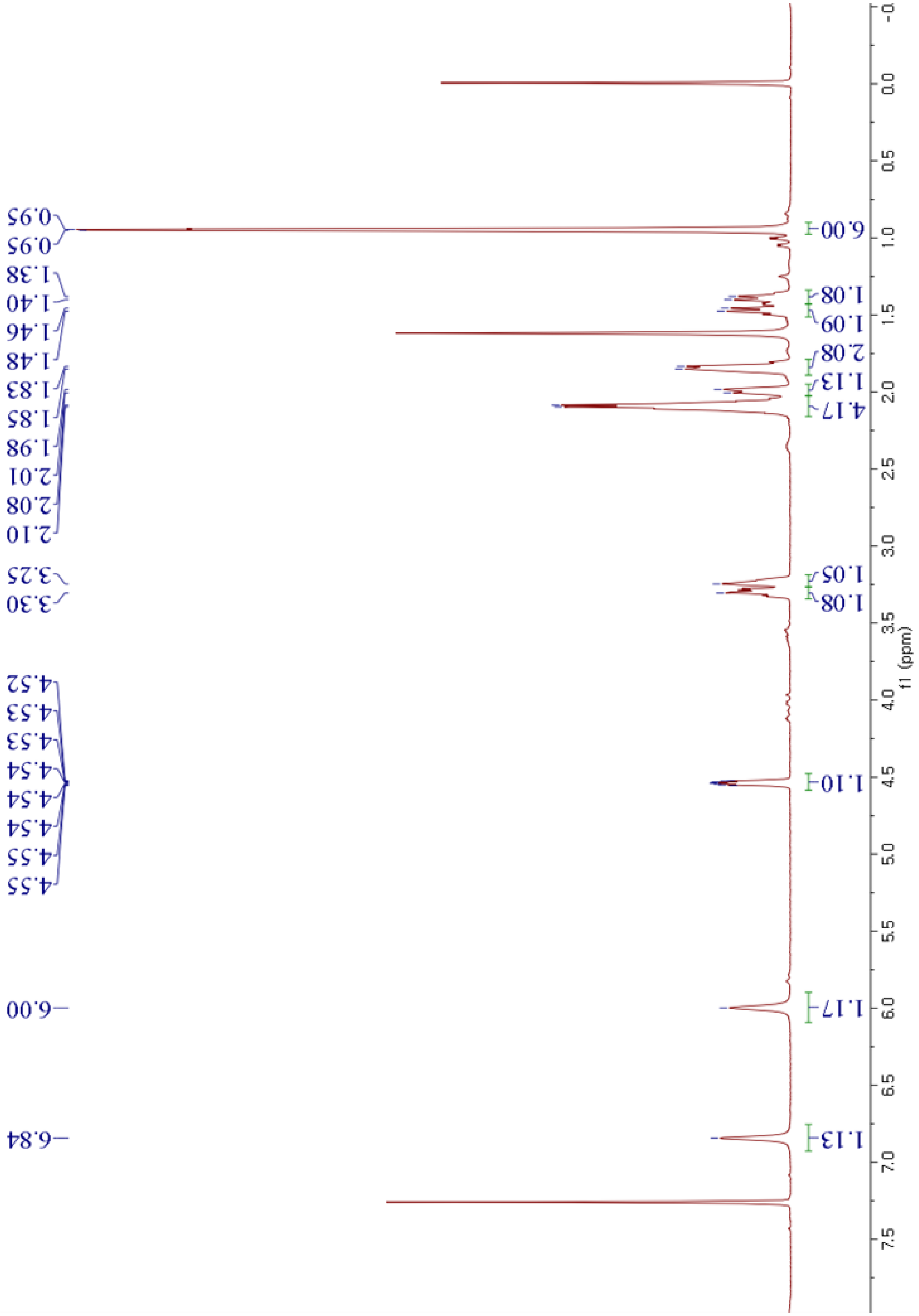

Figure S3. ^{13}C NMR spectrum (150 MHz) of **1** in $CDCl_3$

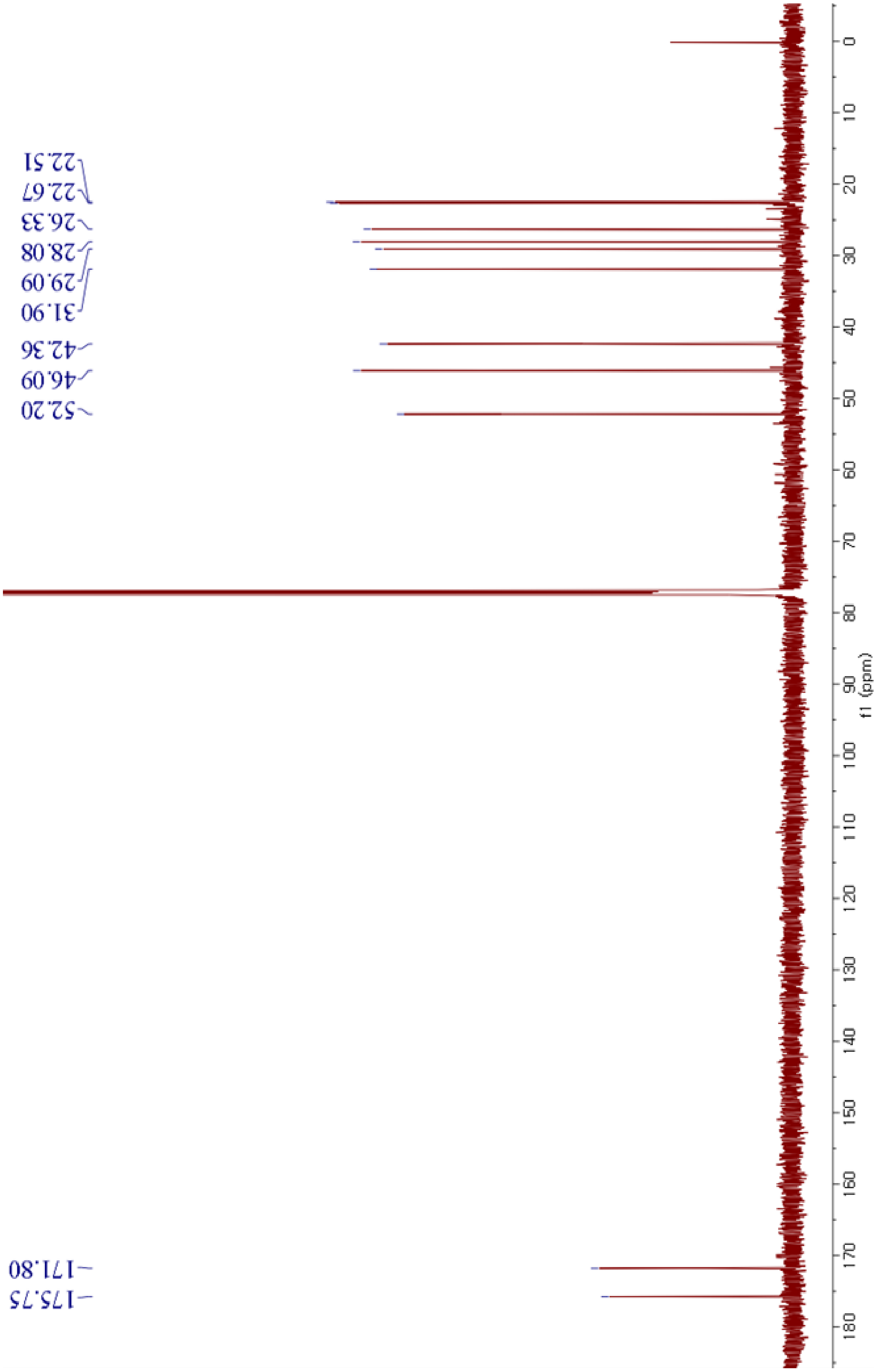

Figure S4. COSY spectrum of **1** in $CDCl_3$

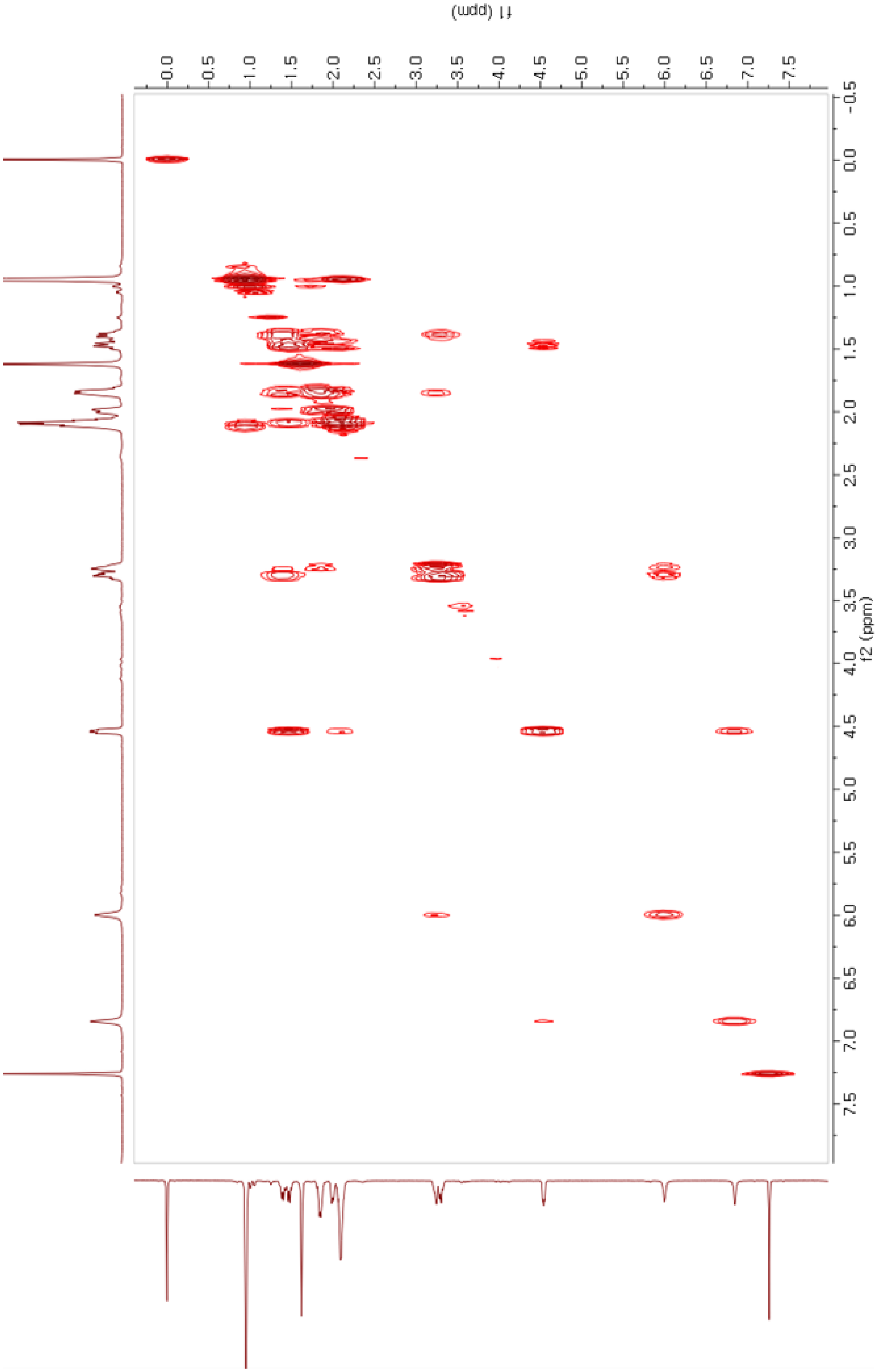

Figure S5. Phase-sensitive HSQC spectrum of **1** in $CDCl_3$

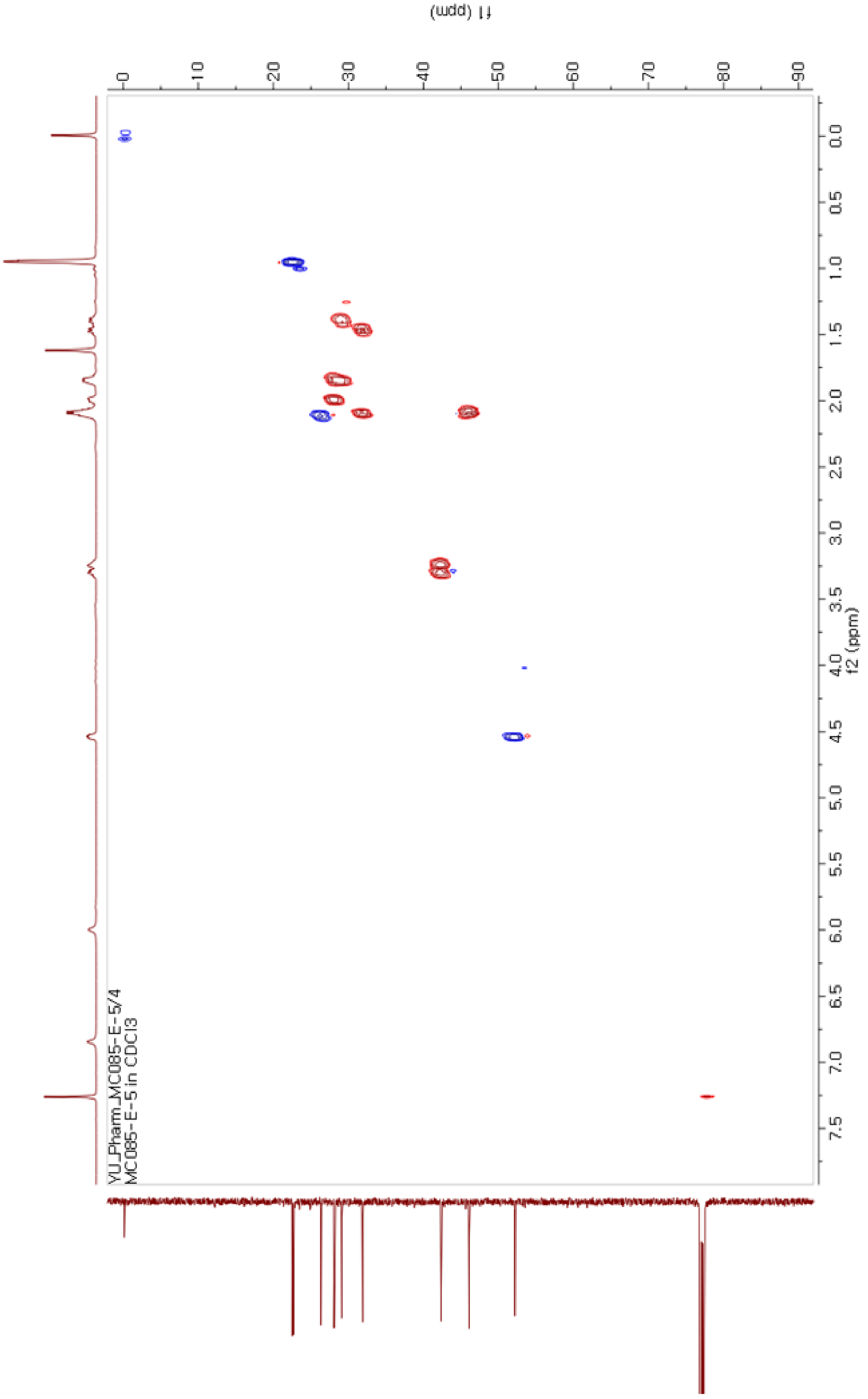

Figure S6. HMBC spectrum of **1** in $CDCl_3$

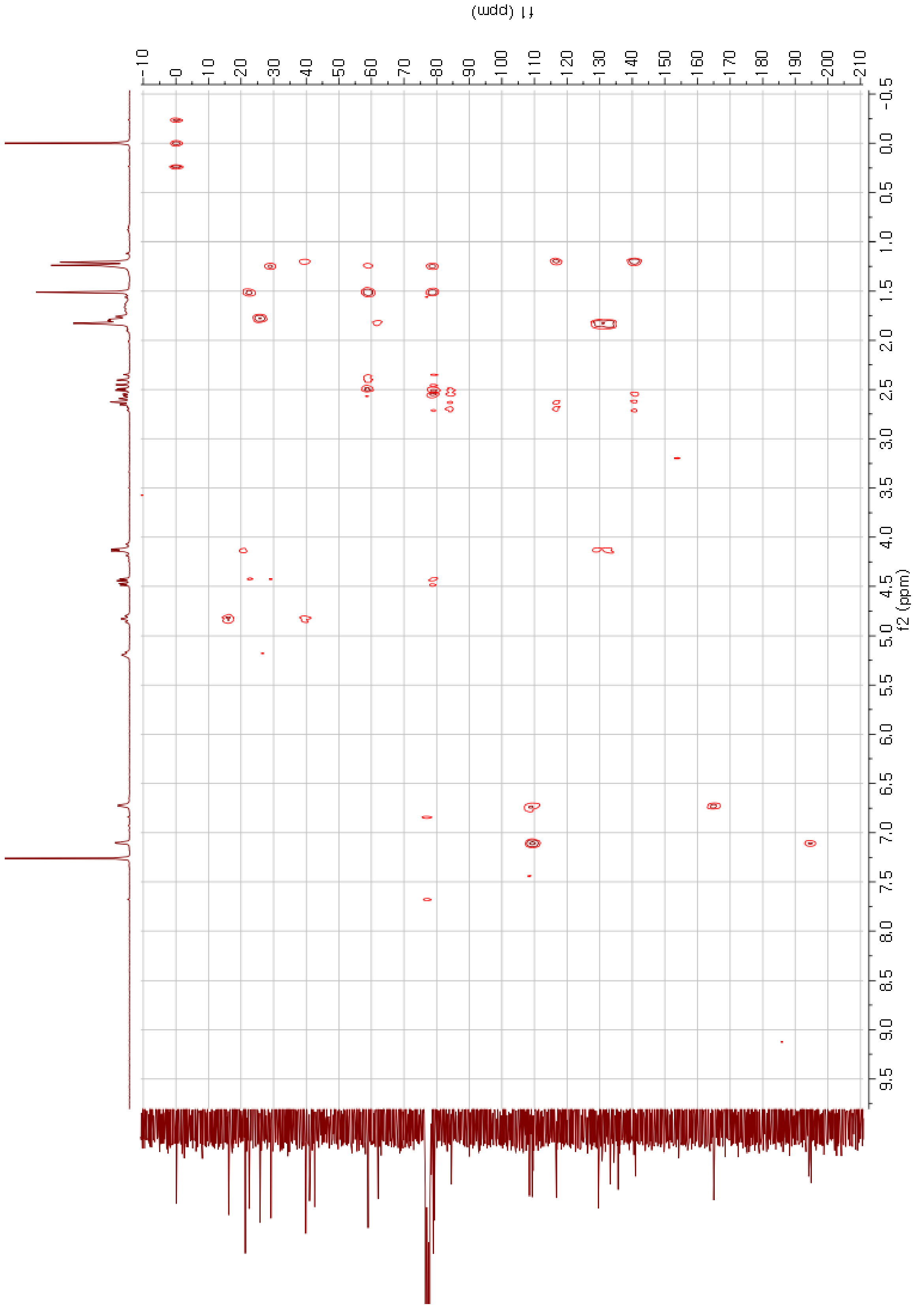

Figure S7. Chiral separation of synthetic compounds **1** (synthetic caprolactam 2) and **2** (synthetic caprolactam 1)

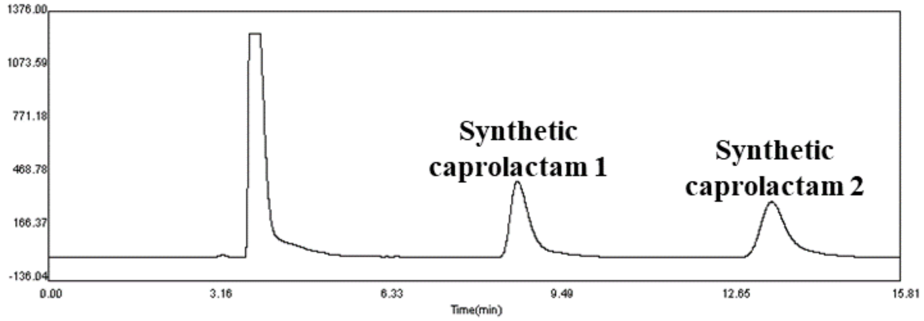

Figure S8. ^{1}H NMR spectrum (250 MHz) of **2** (a synthetic caprolactam 1) in $CDCl_3$

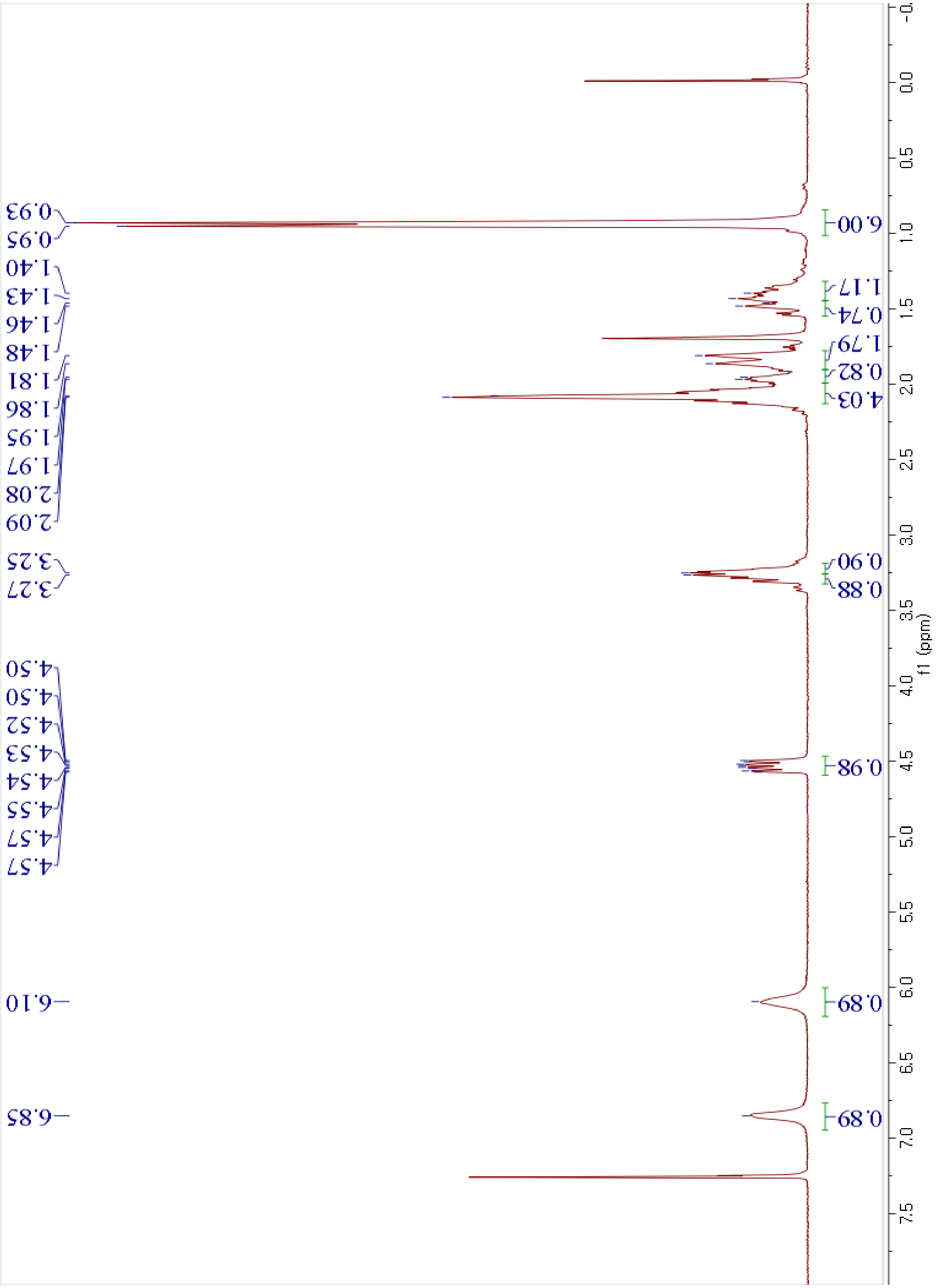

Figure S9. ^{13}C NMR spectrum (63 MHz) of **2** in $CDCl_3$

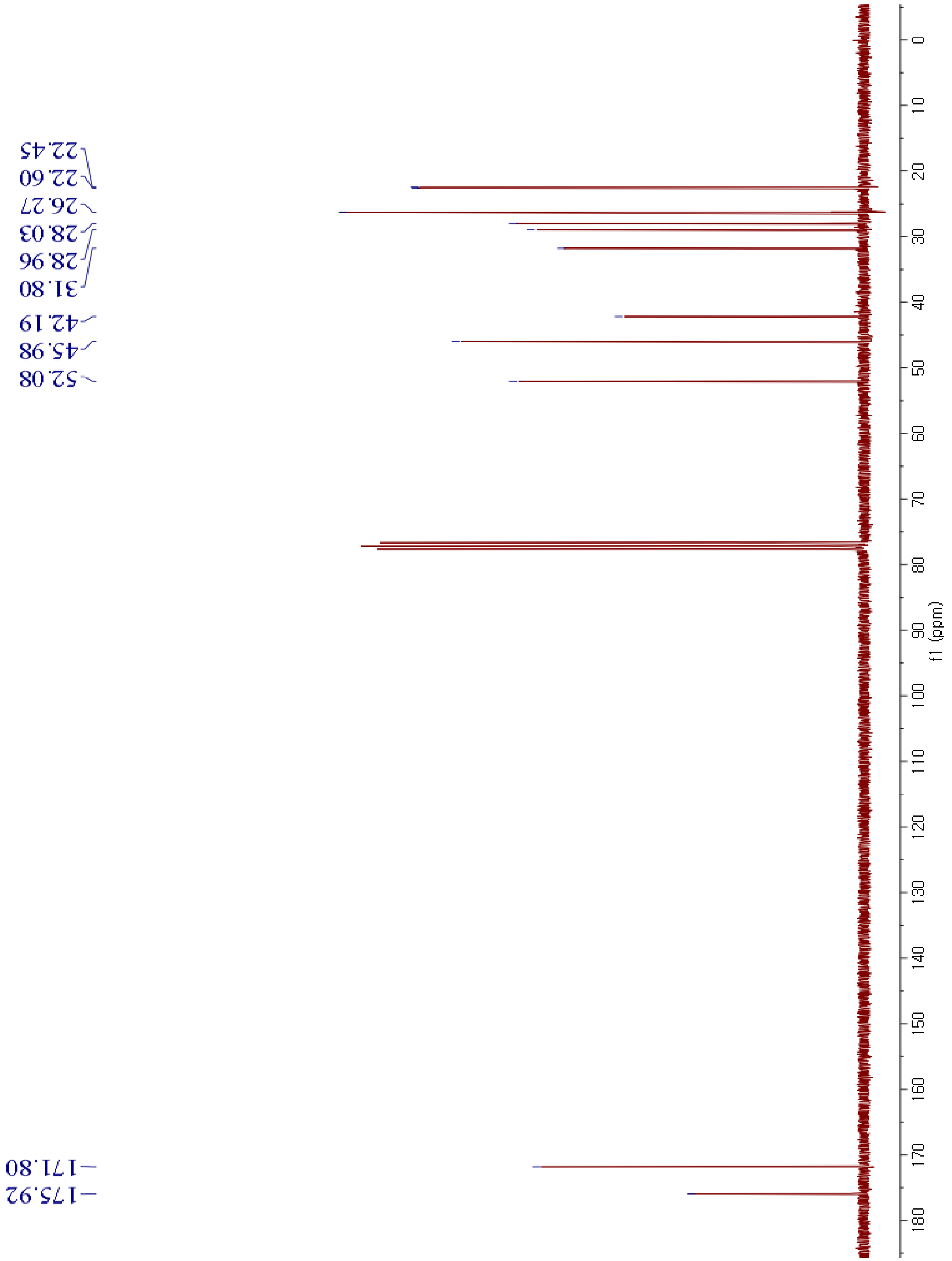

Figure S10. Comparison of ^{1}H NMR spectra of **1** (natural), **1** (synthetic) and **2** (synthetic)

^{1}H-NMR spectrum (600 MHz) of **1** (natural product) in $CDCl_3$

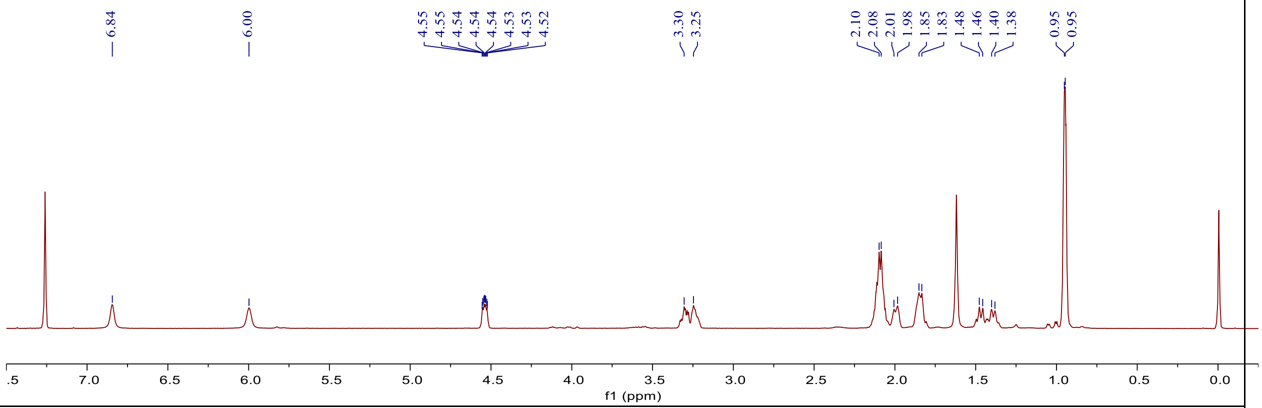

^{1}H-NMR spectrum (250 MHz) of **1** (synthetic product) in $CDCl_3$

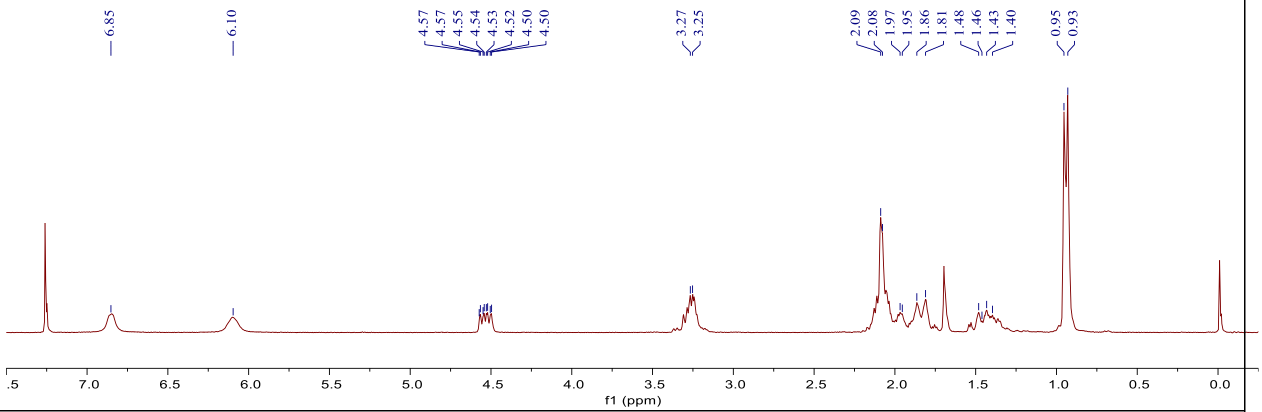

^{1}H-NMR spectrum (250 MHz) of **2** (synthetic product) in $CDCl_3$

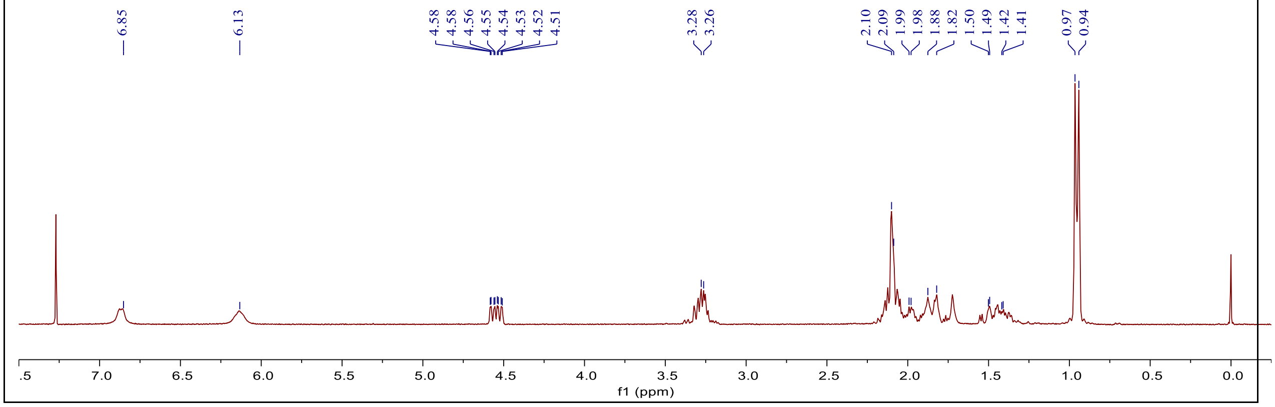

Supplement: Supplementary file 1 [file marinedrugs-19-00465-s001.zip › marinedrugs-1276571-supplementary.pdf]
